# Using Practice Facilitation to Increase Rates of Colorectal Cancer Screening in Community Health Centers, North Carolina, 2012–2013: Feasibility, Facilitators, and Barriers

**DOI:** 10.5888/pcd14.160454

**Published:** 2017-08-17

**Authors:** Bryan J. Weiner, Catherine L. Rohweder, Jennifer E. Scott, Randall Teal, Alecia Slade, Allison M. Deal, Naima Jihad, Marti Wolf

**Affiliations:** 1 Department of Global Health, University of Washington, Seattle, Washington; 2 University of North Carolina at Chapel Hill, Chapel Hill, North Carolina; 3 Philadelphia College of Osteopathic Medicine, Philadelphia, Pennsylvania; 4 North Carolina Community Health Center Association, Raleigh, North Carolina

## Abstract

**Introduction:**

Practice facilitation involves trained individuals working with practice staff to conduct quality improvement activities and support delivery of evidence-based clinical services. We examined the feasibility of using practice facilitation to assist federally qualified health centers (FQHCs) to increase colorectal cancer screening rates in North Carolina.

**Methods:**

The intervention consisted of 12 months of facilitation in 3 FQHCs. We conducted chart audits to obtain data on changes in documented recommendation for colorectal cancer screening and completed screening. Key informant interviews provided qualitative data on barriers to and facilitators of implementing office systems.

**Results:**

Overall, the percentage of eligible patients with a documented colorectal cancer screening recommendation increased from 15% to 29% (*P* < .001). The percentage of patients up to date with colorectal cancer screening rose from 23% to 34% (*P* = .03). Key informants in all 3 clinics said the implementation support from the practice facilitator was critical for initiating or improving office systems and that modifying the electronic medical record was the biggest challenge and most time-consuming aspect of implementing office systems changes. Other barriers were staff turnover and reluctance on the part of local gastroenterology practices to perform free or low-cost diagnostic colonoscopies for uninsured or underinsured patients.

**Conclusion:**

Practice facilitation is a feasible, acceptable, and promising approach for supporting universal colorectal cancer screening in FQHCs. A larger-scale study is warranted.

## Introduction

US federally qualified health centers (FQHCs) have joined the “80% by 2018” initiative sponsored by the National Colorectal Cancer Roundtable, which has the goal of bringing organizations together to screen 80% of adults aged 50 to 75 years for colorectal cancer (CRC) by 2018 (1). To achieve this goal, FQHCs must shift from an opportunistic approach to a universal approach, whereby every eligible patient is offered screening. Although some FQHCs can make this shift on their own, others need support to do so (2). Practice facilitation is a promising strategy for providing such support. Practice facilitation involves sending trained individuals to work with clinic staff to implement office systems (eg, screening reminders, tracking systems, decision aids, communication tools) that help providers use and sustain evidence-based practices (3).

Practice facilitation is effective in improving preventive service delivery and chronic disease management in primary care practices (4). However, its feasibility has not been tested in FQHCs, and whether its effectiveness applies to FQHCs is unclear. Practice facilitation has been tested primarily in private practices, in countries with universal health insurance, or in clinics participating in provider-based research networks (5–16). FQHCs differ from these settings in that they operate in medically underserved communities, see patients with little or no insurance, provide a range of services, and face additional regulatory and funding requirements.

We conducted a pilot study to assess whether practice facilitation could help FQHCs implement office systems needed to shift to universal CRC screening. By using an evidence-informed tool kit (17), a practice facilitator provided assessment, training, technical assistance, and feedback for 12 months to 3 North Carolina FQHC clinics. In addition to reporting changes in recommendation for and completion of CRC screening, we describe facilitators of and barriers to implementing office systems in FQHCs by using the practice facilitation and tool kit approach.

## Methods

### Study design and conceptual model

We used a mixed-methods, one-group, pre/post study design, with the individual FQHC clinic serving as the unit of analysis. This study design is suitable for exploratory or developmental research and is commonly used in feasibility studies (18).

The study was guided by an organizational model of implementation that we developed previously (Figure) (19,20). The model posits that consistent, high-quality delivery or use of an evidence-based practice, such as US Public Health Service CRC screening guidelines (implementation effectiveness), is a function of 1) the office systems that a clinic employs (implementation policies and practices); 2) the technical assistance provided by the practice facilitator (implementation support); and 3) the resulting perception among clinic staff that consistent, high-quality delivery or use of an evidence-based practice is expected, supported, and rewarded (implementation climate). The practice facilitator supports the implementation of office systems and strengthens the implementation climate by reinforcing the priority of universal CRC screening.

**Figure:**
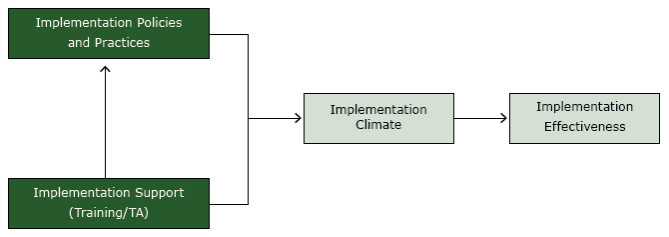
Organizational model of innovation implementation, study on colorectal cancer screening in community health centers, North Carolina, 2012–2013. Abbreviation: TA, technical assistance.

### Study setting

At the time of the study, North Carolina had 26 FQHCs delivering services in 120 clinical sites to nearly 400,000 patients, 70% of whom lived below the federal poverty level, half of whom were uninsured, and 23% of whom were age 50 years or older (21). With assistance from the North Carolina Community Health Center Association, we recruited 3 FQHCs with 5 clinical sites for participation from the 20 that were within a 2-hour driving distance of where the practice facilitator was based and that had an electronic medical record (EMR) system in place. Three FQHCs were selected on the basis of their leadership’s high interest in CRC screening and identification of a “project champion” to ensure that clinics could continue assessment and improvement work between practice facilitator visits. One FQHC with 2 clinical sites dropped out early in the study, preferring to address barriers to CRC screening without assistance from the practice facilitator. Table 1 describes the 3 clinics from the 2 FQHCs that participated in the study.

**Table 1:** Clinic Characteristics, Study on Colorectal Cancer Screening in Community Health Centers, North Carolina, 2012–2013

Characteristic	Clinic, Geographic Location		
	Clinic A, Rural	Clinic B, Rural	Clinic C, Urban
Staffing	2 MDs, 1 PA, 5 RNs, 2 medical assistants, 2 laboratory technicians	1 MD, 1 PA, 1 CNM, 2 LPNs, 1 medical assistant	1 MD, 1 PA, 1 LPN, 1 medical assistant

Patient population, age	773 patients, >50 y	1,884 patients, > 50 y	1,365 patients, >50 y

No. of practice facilitator visits	12	19	18

Average visit duration	Approximately 2 h	Approximately 1 h 45 min	Approximately 1 h

Abbreviations: CNM, certified nurse midwife; LPN, licensed practical nurse; MD, medical doctor; PA, physician assistant; RN, registered nurse.

### Implementation strategy

Starting in April 2012, each clinic received practice facilitation for 12 months from a professional with a master of public health degree with quality improvement experience. As a participation incentive, practices received a cash payment and a supply of immunological fecal occult blood test (iFOBT) kits and mailers. We chose to provide iFOBT kits, because North Carolina does not have state funding for CRC screening for people who are uninsured. During initial visits, the practice facilitator inventoried clinics’ existing resources and office systems for CRC screening and conducted in-service education on the US Public Health Service’s CRC screening guidelines. In subsequent visits, the practice facilitator worked with clinic staff to select and implement policies and procedures from a tool kit developed by the National Colorectal Cancer Roundtable (22) and adapted by the research team for use in FQHCs. The tool kit included evidence-based tools to support universal CRC screening based on US Preventive Services Task Force (USPSTF) guidelines (23). Tool kit components include office systems for CRC screening that fit the practice, its patients, and local conditions; communication systems to support shared informed decision making; and reminder systems to cue providers and patients to take action. The evidence-informed tool kit was created with input from FQHC providers and staff, and the evidence-based practice facilitation model was designed to be flexible and responsive to the needs of the clinics. The research team created additional tools such as a sample clinic self-assessment, screening algorithm, and iFOBT policy; sample counseling scripts and links to patient materials and decision aids; and a sample integrated summary, tracking log, and letters to remind patients to return iFOBT kits. During the 12-month intervention period, clinics received between 12 and 19 visits from the practice facilitator (Table 1). Average visit length ranged from 60 to 120 minutes.

### Data collection and analysis

We conducted pre/post intervention chart audits to determine changes in the percentage of patients with a documented recommendation for CRC screening and who were up to date with screening. We used the USPSTF definition of “up to date,” which is receipt of an iFOBT within the past year or a colonoscopy within the past 10 years (23). We randomly selected 100 charts per clinic for patients aged 50 to 75 years who had not been diagnosed with CRC and had at least 1 visit between January 1, 2011, and October 31, 2011 (preintervention), and June 1, 2013, and March 31, 2014 (postintervention). We abstracted 300 charts preintervention and 255 charts postintervention. The number of charts was not equivalent for the 2 points because one of the clinic sites began to integrate their patient records into the main site’s EMR, whereas previously the 2 systems had been separate. Fisher’s exact tests were used to compare preintervention and postintervention rates within clinics. For overall estimates, generalized estimating equation models with a logistic link were used to perform logistic regression, while accounting for the variability or clustering among patients within separate clinics. Differences among demographic variables such as race/ethnicity, sex, and insurance status were examined.  All analyses were conducted using SAS statistical software, version 9.3 (SAS Institute, Inc).

We also conducted 30-minute semistructured interviews postintervention with 1 to 3 key informants per clinic (N = 6) including administrators and clinicians. We inquired about facilitators of and barriers to implementing office systems changes using the CRC tool kit (implementation policies and practices), satisfaction with the amount and quality of support provided by the practice facilitators (implementation support), and the extent to which systematic CRC screening was expected, supported, and rewarded (implementation climate). Interviews were recorded, transcribed verbatim, and independently coded by 2 qualitative analysts using ATLAS.ti and a starting list of codes keyed to the conceptual framework, supplemented by codes that emerged during analysis.

## Results

Overall, the percentage of eligible patients who received a documented recommendation for CRC screening increased from 15% preintervention to 29% postintervention (*P* < .001). Nonwhite patients were significantly more likely to receive a recommendation during the preintervention period (22% vs 12%, *P* < .001), but this difference did not persist during postintervention (33% vs 27%, *P* = .13). White and nonwhite patients rose to similar levels of screening recommendations. No differences were seen in the rate of recommendation between insurance categories. 

The difference in receipt of a recommendation at postintervention compared with preintervention remained significant, even after controlling for race and insurance (odds ratio [OR] = 2.28; 95% confidence interval [CI], 1.36–3.82; *P* = .002). Clinic A experienced a significant 25 percentage point absolute increase in screening recommendations (12% to 37%, *P* < .001), and Clinic C experienced a 15 percentage point absolute increase (23% to 38%, *P* = .06) (Table 2). With the assistance of the practice facilitator, clinics transitioned from primarily referring patients for screening colonoscopy, which many patients could not afford, to primarily offering iFOBT first. Across all 3 clinics, the percentage who received a recommendation for iFOBT as opposed to colonoscopy increased from 20% preintervention to 64% postintervention (*P* < .001).

**Table 2:** Effects of Practice Facilitation on CRC Screening Rates, Recommendations for Screening, and Recommendations for iFOBT, Study on CRC Screening in Community Health Centers, North Carolina, 2012–2013

Clinic Name (Pre/Post No.)	Preintervention, % (n)	Postintervention, % (n)	Percentage Point Change	*P* Value
**Patients up-to-date with CRC screening**				
A (n = 100; n = 100)	13 (13)	35 (35)	22	<.001
B (n = 100; n = 100)	32 (32)	35 (35)	3	.76
C (n = 100; n = 55)	23 (23)	33 (18)	10	.25
All (n = 300; n = 255)	23 (68)	34 (88)	11	.03
**Patients who received documented CRC screening recommendation**				
A (n = 100; n = 100)	12 (12)	37 (37)	25	<.001
B (n = 100; n = 100)	11 (11)	16 (16)	5	.41
C (n = 100; n = 55)	23 (23)	38 (21)	15	.06
All (n = 300; n = 255)	15 (46)	29 (74)	14	<.001
**Patients recommended for iFOBT screening**				
A (n = 12; n = 37)	33 (4)	65 (24)	32	.09
B (n = 11; n = 16)	0	63 (10)	63	.001
C (n = 23; n = 21)	22 (5)	62 (13)	40	.01
All (n = 46; n = 74)	20 (9)	64 (47)	44	<.001

Abbreviations: CRC, colorectal cancer; iFOBT, immunochemical fecal occult blood test.

Overall, the percentage of patients who were up to date with CRC screening rose from 23% preintervention to 34% postintervention (*P* = .03). In Clinic A, the percentage rose significantly from 13% preintervention to 35% postintervention (*P* < .001). In Clinic C the percentage rose from 23% to 33%, but the change was not significant (*P* = .25) (Table 2). There were no differences by race/ethnicity, sex, or insurance status.

### Facilitators and barriers

All 3 clinics implemented office systems changes to support universal screening such as reminders, tracking logs, and referral systems. The fact that the evidence-informed tool kit was created with input from FQHC providers and staff likely enhanced its usability and effectiveness. Key informants in all 3 clinics cited the implementation support from the practice facilitator as critical for initiating new office systems or improving existing ones. One provider illustrated this point by saying,

[She] has helped us to set up some tracking processes and made sure that we get it [the iFOBT kit] back. That’s been another problem: giving the kit, and it doesn’t come back. She helped us to develop processes to get those patients contacted, so we can get the kit back or if they lost it they can get another one. And there have been a few cases of saved colon cancer. [Clinic B, MD, male]

Assistance with reprogramming data fields in the EMR and back-entering CRC screening information was the most time-intensive, yet valuable, role for the practice facilitator, given the importance of EMRs in monitoring the quality and success of screening programs.

Key informants also reported that the tool kit complemented on-site technical assistance, although some tools were more helpful than others. Providers found useful the clinic self-assessment survey, the sample screening algorithm, the standing orders for CRC screening, the tracking log, and the patient reminders. Providers found less useful the patient counseling scripts (too long), the patient decision aides (too time-consuming), and the integrated summary (paper-based, not EMR-ready). Key informants appreciated flexibility in how they implemented tools. For example, all 3 clinics initially used the HIPAA (Health Insurance Portability and Accountability Act)–compliant postcard reminders included in the tool kit but later switched to a letter that could be loaded into the EMR.

Key informants in all 3 clinics noted that modifying the EMR was the biggest challenge and most time-consuming aspect of implementing office systems changes. One of the first tasks for the practice facilitator was to reprogram the reminders so that they did not keep showing up after the screening had been completed. Another task was to make sure that the new reminders were scheduled with appropriate intervals for screening. One participant described the importance of shifting from tracking distribution of iFOBT kits on paper to tracking in the EMR:

We are going to have to find work arounds to augment what the EMR does to help us achieve the outcomes that we want to for our patients . . . because it is superfluous to have 15 different logs in a 1-inch binder, and you’re flipping through to find whichever one you need to. [Clinic A, MD, male]

Although none of the EMRs had ideal tracking and reminder functions, clinic personnel realized that automated systems are critical in adhering to CRC screening guidelines.

Other barriers included staff turnover, which caused clinics to redistribute staff roles and train new staff in the clinic’s office systems, and reluctance on the part of local gastroenterology (GI) practices to perform free or low-cost diagnostic colonoscopies for uninsured or underinsured patients. One clinic had agreements with local GI practices; the other 2 did not. Despite repeated attempts, these clinics could not get local GI physicians to offer discounted care and had to make arrangements with an academic medical center 2 hours away.

The implementation support that clinics received and the office systems changes they implemented produced a positive implementation climate for universal CRC screening:

. . . I think we learned a lot, we’ve improved our screening numbers, and I think our patients are more engaged. . . . I know my staff and providers are aware of it now and doing a better job. And more importantly for me, I think we are developing systems now to make sure that we do a better job with the follow up. [Clinic B, MD, male]

Key informants from all 3 clinics noted that CRC screening is now considered a top priority and, in one clinic, CRC screening is discussed in every staff meeting. Implementing standing orders empowered nurses to give screening tests to patients and explain how to use them before the provider enters the exam room. Assigning medical assistants and front desk staff responsibility for maintaining the tracking log and patient reminders made the clinic’s effort to institute universal CRC screening a “team sport.” In one clinic, however, key informants acknowledged that clinic administration could have done more upfront to set expectations for staff, make time to work with the practice facilitator, and implement office systems changes. Key informants at another clinic noted that competing priorities for both providers and patients had acted as a “double whammy” that limited the frequency with which providers recommended screening and patients adhered to the recommendation.

## Discussion

The results of this pilot study indicate that practice facilitation is a feasible, acceptable, and promising approach for assisting FQHCs to implement office systems changes that support universal CRC screening. In 3 recent studies of practice facilitation and CRC screening, 2 had no significant results (7,24), and one resulted in a 16% increase in screening rates in the intervention group (25). In our study, which took place exclusively in FQHCs, we achieved an increase of 11 percentage points in patients who were up to date with screening. Although a larger scale test of this approach in the FQHC environment is needed, our results suggest that the practice facilitation strategy can be extended from the private practice setting to FQHCs despite differences in patient populations served, scope of services provided, and funding and regulatory requirements. Practice facilitation is a more intensive, and therefore more costly, approach to implementing office systems changes than “lighter touch” approaches, such as provider education or expert consultation. Adaptive trial designs such as the Sequential Multiple Assignment Randomized Trial (SMART) (26) could be useful for generating evidence about how to modulate the intensity of practice facilitation based on FQHCs’ early responsiveness to the approach. Responsiveness measures could include the level of provider engagement, number of plan–do–study–act cycles completed, or number of office systems changes implemented.

Qualitative results offer insights into why some of the participating clinics were more successful than others. The clinic that experienced the most significant improvements had a nurse who was actively engaged with the practice facilitator, even though she was not originally identified as being the project champion. She changed general clinic policies and took charge of the iFOBT kit distribution without significant effort on the part of the physicians. This clinic was also transitioning from paper charts to an EMR system, which provided an opportunity to create screening documentation and reminders for all eligible patients as their records went online. The second clinic that demonstrated improvement was a single-provider office, and the physician showed a keen interest in working with the practice facilitator to update all of her patient records to accurately reflect CRC screening activities and completion rates. Because the provider had a high degree of control in terms of ensuring that changes that were implemented (eg, distributing a patient survey to get feedback on the type of iFOBT kit that was being used), there were very few barriers to adopting strategies from the tool kit. The third clinic in our sample did not engage in the same high level of implementation activities, which the practice facilitator attributed to a “disconnect” between the quality improvement coordinator with whom she worked and the 2 clinicians who were directly responsible for recommending screening and distributing iFOBT kits. This same clinic, however, began the study without any type of iFOBT program in place and relied solely on referring insured patients for colonoscopies. They did succeed in implementing policies and procedures for including iFOBT kits as a screening option, which represents a major shift in clinical care. These qualitative results suggest that leadership, readiness, communication, and other features of the “inner context” (27) can moderate the effectiveness of practice facilitation in promoting office systems changes for universal CRC screening.

A lesson learned concerns the organizational level at which the practice facilitator intervened. FQHCs often have multiple clinical sites; we chose to implement the intervention and collect data at the clinic level rather than the FQHC level. This approach worked well for 2 FQHCs, but the FQHC that stopped participating did so because CRC screening activities were generally managed at headquarters rather than individual clinics. When the practice facilitator attempted to identify project champions and make site visits, it became clear that providers and staff had little to do with CRC screening office systems. Quality improvement team members at the lead organization were responsible for tasks such as identification of patients due for screening, making referrals for colonoscopies, and ensuring transportation for colonoscopies. As the study progressed, leadership did not feel that practice facilitation would add value to their existing efforts. Like private practices, many FQHCs are joining accountable care organizations or affiliating with health care systems (28). Future research should account for varying degrees of organizational embeddedness of clinical sites. Practice facilitation may need to implement multilevel strategies that target higher organizational levels to create an enabling, supportive context for office systems changes at the clinic level.
